# Academic involution atmosphere and AI dependence among university students: the mediating role of academic stress and the moderating role of individual academic involution behaviors

**DOI:** 10.3389/fpsyg.2026.1857123

**Published:** 2026-06-08

**Authors:** Fangda Fu

**Affiliations:** School of Drama, Film, and Television, Communication University of China, Beijing, China

**Keywords:** academic involution atmosphere, academic stress, AI dependence, individual academic involution, social comparison

## Abstract

With the rapid development of generative AI, its deep integration into university students’ learning and daily life has made AI dependence increasingly prevalent. While existing studies have primarily focused on individual psychological mechanisms of AI dependence, they have paid less attention to the role of external social environments, particularly the increasingly prevalent phenomenon of academic involution in higher education. Guided by the I-PACE framework and integrating stress–coping theory, social comparison theory, and conservation of resources theory, we propose that academic involution atmosphere relates to students’ AI dependence through academic stress, with individual academic involution behavior moderating this association. Using PLS-SEM to analyze data from 500 Chinese university students, we find that academic involution atmosphere is associated with higher academic stress. Higher academic stress is associated with students viewing AI as a technological substitute, which correlates with greater AI dependence. Higher individual involution attenuates the positive association between involution atmosphere and academic stress. This moderated mediation model offers insights into the associations among these variables, providing insights for universities to alleviate stress, optimize the competitive atmosphere, and guide rational AI use.

## 1 Introduction

The rapid development of artificial intelligence (AI) is profoundly revolutionizing teaching and learning in higher education. As students increasingly adopt AI tools in learning, some may develop stronger reliance on these technologies in academic contexts. Following Zhang et al. (2024), AI dependence is conceptualized as a context-specific form of problematic technology use (PTU) characterized by excessive reliance on AI tools, psychological attachment to them, and perceived difficulty functioning effectively without AI support. Unlike traditional forms of PTU such as internet or social media addiction (Yang et al., 2021), AI dependence in educational settings is primarily task-oriented and associated with learning efficiency and academic task completion rather than entertainment or hedonic gratification. Existing studies have explored both the consequences (Mukhtar et al., 2025; Zhang et al., 2025) and antecedents (Bukhari et al., 2025; Li et al., 2025; Ye et al., 2025). However, existing research has primarily focused on individual-level psychological factors, providing limited insight into how external academic environments may be associated with students’ reliance on AI tools.

The Interaction of Person-Affect-Cognition-Execution Model (I-PACE) provides an integrative theoretical framework for understanding technology-related dependence and problematic technology use (Brand et al., 2019). The model suggests that such behaviors are associated with interactions among person factors, affective responses, cognitive processes, and executive functions. Consistent with this framework, prior research has identified numerous individual-level factors associated with AI dependence, including anxiety (Hu et al., 2023; Huang et al., 2024), perceived usefulness (Liu, 2025; Samant et al., 2024), and self-control (Li et al., 2025; Ye et al., 2025). In the present study, AI dependence is conceptualized as a function-oriented and coping-related reliance on AI tools in academic contexts rather than a primarily entertainment-oriented form of technology use. However, prior research drawing on the I-PACE framework has tended to focus primarily on individual psychological mechanisms, with comparatively less attention to the broader social environment in which technology use occurs. In higher education, one salient contextual phenomenon is academic involution, which refers to a situation in which individuals invest increasing effort in competition despite diminishing returns (Liu et al., 2022; Zhou et al., 2022). Importantly, academic involution can be understood at both the environmental and individual levels. At the environmental level, students may perceive a pervasive academic involution atmosphere (intense peer competition and escalating academic effort); at the individual level, they may engage in behaviors like increasing effort to outperform peers. Guided by selected concepts from the I-PACE framework, the present study examines how an external academic context, namely academic involution atmosphere, is associated with students’ psychological responses associated with AI dependence.

Within this overarching framework, the proposed moderated mediation model primarily draws on stress-coping theory. Academic involution atmosphere, as an environmental stressor, may be positively associated with academic stress, which may relate to greater reliance on AI as a coping resource. Drawing on social comparison theory, competitive environments may be associated with greater awareness of performance gaps, which may relate to perceived academic demands and may be associated with greater reliance on AI tools. Individual academic involution moderates this process: students with higher self-regulated learning and engagement may experience less stress under the same environment. Theoretically, this study integrates social environment and individual behavior perspectives to explain the association between competitive academic environments and students’ AI dependence, drawing on insights from the I-PACE framework to highlight the link between academic involution atmosphere, psychological responses, and technology use. Practically, the findings provide implications for universities to support students in coping with competitive pressures and promoting responsible AI use.

## 2 Literature review and theoretical hypotheses

### 2.1 Academic involution atmosphere and AI dependence

“Involution” refers to a state in which individuals engage in mutual competition and internal consumption for limited resources, akin to “malignant competition,” resulting in a continuously decreasing personal “effort-to-reward ratio” (Geertz, 1963). In recent years, the expansion of higher education has increased graduate numbers and intensified job-market competition. To enhance employability, students have invested more in academics, fostering stronger competition. Students compete for scarce resources (e.g., scholarships, graduate recommendations) and increase engagement in coursework and activities. Due to resource scarcity and fixed competition mechanisms, this collective effort evolves into academic involution (Zhou et al., 2022). Academic involution atmosphere refers to an environment where students intensify efforts to pursue limited opportunities, with escalating input yielding diminishing marginal returns, rising standards, and an imbalance between effort and outcome (Liu et al., 2022; Zhou et al., 2022).

Consistent with Zhang et al. (2024), AI dependence in this study refers to excessive functional reliance on AI technologies for academic tasks, accompanied by psychological attachment and perceived difficulty completing learning activities independently of AI support. Moreover, social comparison theory (Festinger, 1954) suggests that individuals evaluate their abilities and performance by comparing themselves with others. In highly competitive academic environments, students are more likely to engage in upward social comparison, focusing on peers who perform better or achieve more. Such comparisons may highlight discrepancies between one’s own performance and that of others, which may be associated with stronger concerns about falling behind and increasing perceived pressure. These comparison processes may also shape students’ perceptions of whether their available resources are sufficient to meet competitive demands.

From the perspective of conservation of resources theory (Hobfoll, 1989), individuals are motivated to protect and acquire resources when facing potential resource loss. When students perceive a gap between their current resources and academic demands, they may seek external means to compensate for such deficiencies. AI technologies, which provide rapid information integration, personalized feedback, and task support (Su and Yang, 2023), may function as a form of resource substitute that is associated with conserving cognitive resources (Gkintoni et al., 2025) and time (Owan et al., 2023). In this sense, greater reliance on AI may be associated with higher levels of AI dependence, both instrumentally and psychologically. Taken together, social comparison processes and resource-based considerations jointly suggest that competitive academic environments may be associated with students’ increased reliance on AI tools. Based on the above reasoning:

*H1*: Academic involution atmosphere is positively associated with university students’ AI dependence.

### 2.2 Academic involution atmosphere and academic stress

Stress is a psychological state resulting from the appraisal of one’s ability to cope with external demands (Lazarus and Folkman, 1984). Academic stress refers to psychological burden and tension when academic demands exceed available resources (Kristensen et al., 2023), typically arising from workload, competition, and student-teacher interactions (Chawla and Sachdeva, 2018). According to social comparison theory (Festinger, 1954), academic involution atmosphere may function as a situational stressor that is associated with greater social comparison, outcome uncertainty, and higher levels of stress. In a highly competitive environment, students frequently compare themselves with peers for scarce resources (Liu et al., 2025). A stronger involution atmosphere reduces the chance of obtaining resources and strengthens relative deprivation (Liu et al., 2022), which may relate to perceptions that demands exceed available resources and may be associated with greater psychological burden (Ni et al., 2024) and responses (Liu et al., 2022). Based on the above, the following hypothesis is proposed:

*H2*: The academic involution atmosphere is positively associated with academic stress.

### 2.3 Academic stress and AI dependence

Under conditions of high academic stress, individuals are likely to adopt both problem-focused and emotion-focused coping strategies to manage academic demands. In this process, generative artificial intelligence (AI) tools may serve as an accessible coping resource by providing instant feedback, information integration, and writing support (Baidoo-Anu and Ansah, 2023). Such functions are associated with improved task efficiency and may be perceived by students as instrumental support for learning. Consistent with this view, prior research has shown that AI-assisted learning is associated with reduced anxiety and improved academic performance when used as a functional educational tool (Sun and Zhou, 2024). However, it is important to distinguish between instrumental AI utilization and excessive reliance on AI tools. While instrumental use refers to goal-directed and controlled engagement with AI to enhance learning outcomes, repeated reliance on AI as a primary means of task completion may be associated with stronger psychological attachment to AI and reduced perceived capability when functioning without such tools.

Following Zhang et al. (2024), the present study conceptualizes AI dependence as a coping-oriented reliance on AI systems in academic contexts. Unlike instrumental AI utilization, which may facilitate learning efficiency and task completion, AI dependence reflects stronger psychological reliance on AI as a coping resource under academic demands. This distinction is important for understanding the boundary between adaptive AI-supported learning and excessive reliance on AI tools in educational contexts.

From a stress–coping perspective, higher levels of academic stress may be associated with greater reliance on AI tools for managing academic demands. When students increasingly rely on AI as a primary coping resource for academic tasks, such reliance may be reflected in higher levels of AI dependence. Accordingly, the following hypothesis is proposed:

*H3*: Academic stress is positively associated with AI dependence.

Based on H1–H3, the overly competitive environment reflected in academic involution atmosphere may be associated with higher academic stress, which may also be associated with greater AI dependence. Therefore, the following hypothesis is proposed:

*H4*: Academic stress mediates the association between academic involution atmosphere and AI dependence.

### 2.4 Moderating role of individual academic involution

Individual academic involution refers to students’ relatively high investment in academic competition and pursuit of educational resources (Zhou et al., 2022). Drawing on the stress–coping framework (Lazarus and Folkman, 1984), environmental stressors are cognitively appraised as either a threat or a challenge, which co-occurs with psychological (McCrae, 1984). Students with higher levels of individual academic involution often exhibit stronger self-regulated learning (Arabzadeh et al., 2012) and academic engagement (Chen et al., 2024), which may function as personal resources associated with more effective coping with academic demands. From this perspective, high individual academic involution may be linked to a greater tendency to adopt problem-focused coping strategies (Kristensen et al., 2023). Such coping-oriented engagement may be associated with more adaptive interpretations of competitive academic environments (Carver et al., 1989), thereby weakening the extent to which academic involution atmosphere is appraised as stressful. In contrast, students with lower levels of individual academic involution may have fewer available coping resources and may therefore be more likely to perceive competitive environments as stressful. Thus, we propose:

*H5*: Individual academic involution negatively moderates the positive association between academic involution atmosphere and academic stress.

**Figure 1:**
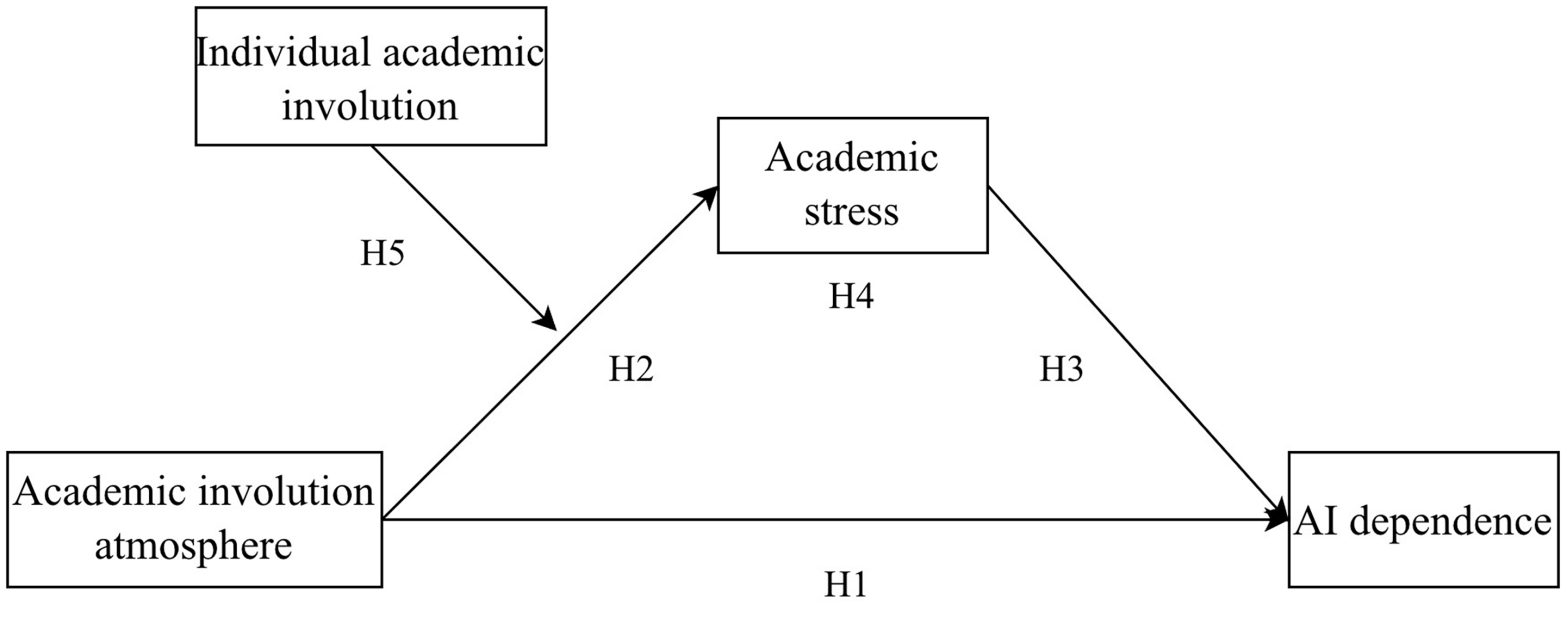
The proposed theoretical framework of the study.

The overall research framework is illustrated in Figure 1.

## 3 Methods

### 3.1 Data source and sample description

This study collected data using a questionnaire survey administered through the online platform Credamo, which functions similarly to Amazon Mechanical Turk. Credamo provides access to a diverse pool of over three million qualified respondents with varied demographic characteristics, covering different genders, age groups, occupations, and regions. The platform has been widely used in academic research on university students’ mental health and AI usage (Yang et al., 2023; Zhang and Yang, 2025). To ensure high-quality responses, each participant was compensated with 3 RMB (approximately 0.43 USD) upon successful completion of the survey. The research protocol was reviewed and approved by the Ethics Committee of the Communication University of China. All procedures involving human participants were conducted in accordance with the ethical standards of the institutional research committee and the 1964 Helsinki Declaration and its later amendments. All participants provided informed consent before beginning the survey and were informed of their right to withdraw at any time. Data collection was conducted from October 11 to 17, 2025, yielding a total of 536 completed questionnaires. Of these, 36 questionnaires were excluded due to failed attention-check items (e.g., “Please select ‘Strongly Disagree’”), resulting in 500 valid responses and an effective response rate of 93.28%. The demographic characteristics of respondents are presented in Table 1.

**Table 1:** Sample demographic characteristics.

Characteristic	Category	Frequency	Percentage (%)
Gender	Female	261	52.2
	Male	239	47.8
Age	18–20	135	27
	21–25	342	68.4
Education level	26 and above	23	4.6
	Junior college	17	3.4
	Undergraduate	391	78.2
	Master’s student	86	17.2
	Doctoral student	6	1.2
Academic discipline	Agriculture	17	3.4
	Medicine	54	10.8
	History	4	0.8
	Engineering	109	21.8
	Education	35	7
	Literature	50	10
	Law	24	4.8
	Science	57	11.4
	Management	96	19.2
	Economics	43	8.6
	Arts	11	2.2
Grade level	First year	42	8.4
	Second year	118	23.6
	Third year	173	34.6
	Fourth year	157	31.4
	Fifth year	10	2
Type of institution	Tier-2 university	140	28
	Tier-1 university	214	42.8
	“211 Project” university	94	18.8
	“985 Project” university	52	10.4

### 3.2 Measurements

The study involves four constructs: academic involution atmosphere, individual academic involution behavior, academic stress, and AI dependence. Except for academic stress, which was measured on a 7-point scale (1 = never, 7 = very often), all other constructs were assessed using 7-point Likert scales ranging from 1 (strongly disagree) to 7 (strongly agree).

Academic involution atmosphere was measured using the scale developed by Zhou et al. (2022), consisting of six items, such as “In my academic environment, students engage in intense competition for academic performance.” and “In my academic environment, many students invest excessive effort in their studies to obtain higher grades.” This scale has been validated and widely used in prior research (Liu et al., 2024; Ni et al., 2024; Wang J. et al., 2025).

Individual academic involution was also measured using the scale developed by Zhou et al. (2022), consisting of six items such as “I often compete intensely with others in academic tasks” and “I try to increase the length of term papers or lab reports to achieve higher grades.” This scale has been widely adopted in previous studies (Liu et al., 2024, 2025).

Academic stress was measured using the academic stress subscale of the stress scale developed by Solberg et al. (1993), consisting of seven items. This subscale captures students’ perceived pressure related to academic tasks or coursework, for example, “I find it difficult to manage a heavy academic workload” and “I have trouble completing course requirements on time.” This scale has been validated in prior research (Torres and Solberg, 2001; Zhao, 2024).

Following Zhang et al. (2024), AI dependence was measured using a five-item scale assessing students’ psychological attachment to AI tools and perceived difficulty functioning independently without AI support in academic contexts, including items such as “I feel anxious when I cannot use AI tools to complete academic tasks” and “Without AI support, I find it difficult to accomplish academic tasks.” This scale captures students’ psychological attachment to AI and perceived difficulty functioning independently of AI tools in academic contexts. The scale has been widely applied in prior research (Chen et al., 2025; Tamrin et al., 2024; Zhong et al., 2024) and emphasizes functional reliance on AI in academic contexts rather than general frequency of AI use or entertainment-oriented technology use.

It is noteworthy that while some scholars operationalize AI reliance through functional or professional lenses, such as perceived usefulness and instructional support (Buniel et al., 2025; Wang X. et al., 2025), the measurement adopted in this study specifically captures problematic psychological reliance on AI in academic contexts. This includes items reflecting anxiety when AI is unavailable and difficulty completing tasks without AI support. This operationalization was chosen to align with our theoretical focus on AI use as a stress-induced compensatory coping mechanism rather than as a purely efficiency-enhancing tool.

This study employed SPSS 29 and SmartPLS 3.2 to assess the reliability and validity of the measurement scales and to test the proposed hypotheses. Variance-based PLS-SEM was employed in this study for several methodological reasons. First, the research model is theory-driven and involves the simultaneous examination of multiple structural relationships, including mediation and moderation pathways among latent constructs. PLS-SEM is well suited for such models as it allows the estimation of complex structural relationships among latent variables while focusing on maximizing the explained variance in endogenous constructs. Second, consistent with the objectives of this study, which emphasize explaining variance in AI dependence through a system of interrelated psychological and contextual factors, PLS-SEM enables the assessment of structural relationships without relying on strict multivariate distributional assumptions or overall model fit indices. This is particularly relevant when the primary interest lies in the relative strength and significance of hypothesized relationships within a theoretically grounded structural model.

This methodological choice is consistent with recent literature suggesting that PLS-SEM is appropriate for theory-driven research when the focus is on structural relationships and explanatory power rather than covariance reproduction (Hair et al., 2019; Rigdon et al., 2017).

## 4 Data analysis and hypothesis testing

### 4.1 Reliability and validity assessment

Although the survey was completed anonymously, all items were answered by the same respondent, which may introduce common method variance (CMV). To address this, Harman’s single-factor test (Podsakoff et al., 2003) was performed for all measurement items. The largest factor accounted for 19.418% of the total variance, well below the 40% threshold, indicating that CMV was not a serious concern. In addition, common method variance was further assessed using the full collinearity approach recommended by Kock (2015). The results indicated that all variance inflation factor (VIF) values ranged from 1.015 to 2.534, well below the recommended threshold of 3.3, suggesting that common method variance was unlikely to pose a substantial threat to the validity of the findings.

The reliability and validity assessment of the measurement model focused on item loadings, internal consistency reliability, convergent validity, and discriminant validity (Hair et al., 2019). All item loadings ranged from 0.784 to 0.913, exceeding the recommended threshold of 0.7, indicating good indicator reliability.

For internal consistency, different scholars suggest slightly different evaluation criteria. Generally, higher reliability indicates better internal consistency. Composite reliability (CR), as proposed by Jöreskog and Sörbom (1993), is commonly used, with values between 0.7 and 0.9 considered satisfactory to good. Values exceeding 0.95 may indicate item redundancy and reduce construct validity (Diamantopoulos et al., 2012). In this study, the four constructs had CR values ranging from 0.919 to 0.948, all within the acceptable range. Cronbach’s *α*, another measure of internal consistency, had values between 0.895 and 0.934 for the four constructs, suggesting good reliability, although this method may provide conservative estimates. To address potential over- or underestimation, ρA was also reported as an improved measure of internal consistency (Dijkstra and Henseler, 2015), with values ranging from 0.905 to 0.951 for all constructs. Collectively, these results indicate satisfactory reliability for all scales.

Convergent validity was assessed using the average variance extracted (AVE), with a minimum acceptable value of 0.50 (Hair et al., 2019). As shown in Table 2, all constructs had AVE values ranging from 0.655 to 0.774, exceeding the 0.50 threshold, indicating good convergent validity.

**Table 2:** Reliabilities, convergent validities and discriminant validities.

Variable	Cronbach’s *α*	rho_A	CR	AVE	AID	IAI	AIA	AS
AI dependence	0.927	0.929	0.945	0.774	**0.880**	0.098	0.176	0.649
Individual academic involution	0.934	0.951	0.948	0.751	0.089	**0.866**	0.634	0.210
Academic involution atmosphere	0.895	0.905	0.919	0.655	0.166	0.578	**0.809**	0.311
Academic stress	0.908	0.911	0.929	0.686	0.598	0.198	0.287	**0.828**

AID, AI dependence; IAI, individual academic involution; AIA, academic involution atmosphere; AS, academic stress. The lower-left triangle presents the inter-construct correlations, the upper-right triangle presents the HTMT values, and the diagonal elements (presented in bold) represent the square roots of the AVEs.

Discriminant validity was assessed by comparing the square root of the AVE for each construct with its correlations with other constructs. If the square root of the AVE is greater than all inter-construct correlations, discriminant validity is considered satisfactory. As shown in Table 2, the diagonal values represent the square roots of the AVE, all of which exceed the correlations with other constructs, indicating adequate discriminant validity. More recently, Hair et al. (2019) recommended evaluating discriminant validity using the heterotrait–monotrait ratio of correlations (HTMT) (Henseler et al., 2015). For models with highly conceptually similar constructs, the HTMT threshold should be below 0.90; for constructs with higher conceptual distinctiveness, a stricter threshold of 0.85 is recommended (Henseler et al., 2015). As shown in Table 2, the HTMT values in the upper-right triangle range from 0.098 to 0.634, all below 0.85, indicating good discriminant validity among the constructs.

### 4.2 Measurement invariance testing

To address potential heterogeneity resulting from the diverse academic levels and disciplines within the sample, we conducted a Measurement Invariance of Composite Models (MICOM) procedure following Henseler et al. (2016). The purpose of this analysis was to assess whether the constructs were interpreted consistently across different sub-groups. Due to the relatively small number of junior college students and doctoral students, participants were regrouped into two broader academic-level categories for the MICOM analysis: undergraduate-level students (including junior college and undergraduate students) and postgraduate-level students (including master’s and doctoral students). In addition, to address potential disciplinary heterogeneity and ensure adequate sample sizes across groups, academic disciplines were classified into STEM and non-STEM categories. STEM disciplines included Agriculture, Medicine, Engineering, and Science, whereas non-STEM disciplines included Education, Literature, Law, Management, Economics, Arts, and History. The MICOM results are presented in Table 3.

**Table 3:** The results of MICOM analysis.

Grouping variable	Construct	Step 2: c	Step 2: p	Step 3: Mean Diff. p	Step 3: Var. Diff. p	Invariance level
Academic level	AI dependence	0.999	0.166	**0.022**	0.238	Partial
	Academic stress	0.999	0.205	0.352	**0.004**	Partial
	Individual involution	0.998	0.818	0.799	0.332	Full
	Involution atmosphere	0.996	0.578	0.081	0.536	Full
Academic discipline	AI dependence	1	0.241	0.058	0.150	Full
	Academic stress	0.999	0.110	0.071	0.155	Full
	Individual involution	0.999	0.922	0.448	0.059	Full
	Involution atmosphere	0.999	0.974	**0.003**	**0.025**	Partial

Following Henseler et al. (2016), compositional invariance is established when the original correlation is not significantly different from 1 (*p* > 0.05). Full invariance indicates that equality of means and variances was also supported, whereas partial invariance indicates that only compositional invariance was established. Values presented in bold indicate partial invariance.

The results of Step 2 (Compositional Invariance) supported compositional invariance across all constructs, as the original correlations were not significantly different from 1 (*p* > 0.05). Although some baseline differences were observed in Step 3 (Equality of Means and Variances), the establishment of partial measurement invariance is generally considered sufficient for meaningful group comparisons and pooled-sample analysis (Henseler et al., 2016). Therefore, the pooled-sample analysis employed in this study was deemed appropriate.

### 4.3 Hypothesis testing

After confirming the reliability and validity of all constructs, the structural model, including the control variables of gender, age, education level, academic discipline, grade, and type of institution, was tested using SmartPLS 3.2. Among the control variables, age was negatively associated with AI dependence (*β* = −0.120, *p* < 0.05, *f^2^* = 0.012), whereas education level was positively associated with AI dependence (*β* = 0.162, *p* < 0.05, *f^2^* = 0.020). The remaining control variables did not show significant associations with AI dependence. All results are presented in Table 4.

**Table 4:** The results of structural model.

Hypothesis	Path	Coefficient	SD	*p*-value	2.5%	97.5%	*f^2^*
H1	Academic involution atmosphere → AI dependence *(without mediator)*	0.127	0.044	0.002	0.062	0.218	0.017
H2	Academic involution atmosphere → Academic stress	0.172	0.066	0.010	0.050	0.312	0.013
H3	Academic stress → AI dependence	0.574	0.036	0.000	0.500	0.643	0.460
H4	Academic involution atmosphere → Academic stress → AI dependence	0.099	0.039	0.011	0.029	0.180	Null
H5	Academic involution atmosphere × Individual academic involution → Academic stress	−0.080	0.037	0.032	−0.150	−0.001	0.008
Control variables	Age → AI dependence	−0.120	0.054	0.026	−0.216	−0.008	0.012
	Gender → AI dependence	−0.055	0.037	0.136	−0.129	0.016	0.004
	Academic Level → AI dependence	0.162	0.060	0.007	0.038	0.274	0.020
	Grade → AI dependence	0.030	0.050	0.551	−0.072	0.124	0.001
	Academic Discipline → AI dependence	0.032	0.036	0.375	−0.038	0.104	0.002
	Type of institution → AI dependence	−0.046	0.037	0.221	−0.119	0.028	0.003

Academic involution atmosphere was not significantly associated with AI dependence (*β* = −0.023, *p* > 0.05 *f^2^* = 0.001). However, academic involution atmosphere was significantly positively associated with academic stress (*β* = 0.172, *p* < 0.01, *f*^2^ = 0.013), indicating an effect size below the conventional threshold for a small effect (Cohen, 1988). Academic stress was significantly positively associated with AI dependence (*β* = 0.574, *p* < 0.001, *f*^2^ = 0.460), indicating a large effect size. In addition, academic stress significantly mediated the association between academic involution atmosphere and AI dependence (*β* = 0.099, *p* < 0.05). The interaction term between academic involution atmosphere and individual academic involution was significantly negatively associated with academic stress (*β* = −0.080, *p* < 0.05, *f*^2^ = 0.008). Although the interaction effect size was relatively small, this magnitude is comparable to typical moderation effects observed in social and behavioral research (Aguinis et al., 2005). Furthermore, the Stone–Geisser’s *Q*^2^ values for academic stress (*Q*^2^ = 0.059) and AI dependence (*Q*^2^ = 0.291) were both greater than zero, indicating that the model demonstrated acceptable predictive relevance (Hair et al., 2019).

To further examine the relationship proposed in H1, a separate model without the mediator was estimated. The results showed that academic involution atmosphere was positively associated with AI dependence before including academic stress (*β* = 0.127, *p* < 0.05, *f^2^* = 0.017). However, after academic stress was introduced into the model, the direct association became non-significant, suggesting that the relationship between academic involution atmosphere and AI dependence primarily operates through academic stress. The indirect association between academic involution atmosphere and AI dependence through academic stress was significant (*β* = 0.099, *p* < 0.01), and the 95% bootstrapped confidence interval did not include zero (95% CI [0.029, 0.180]). After including the mediator, the direct association between academic involution atmosphere and AI dependence was no longer statistically significant (*β* = −0.022, *p* = 0.570). The Variance Accounted For (VAF) exceeded the 80% threshold, and the non-significant direct association is consistent with an indirect-only mediation pattern (Hair et al., 2019).

To explore the moderating role of individual academic involution, a simple slope analysis was conducted. As shown in Table 5, for students with low levels of individual academic involution (M-1SD), academic involution atmosphere was positively and significantly associated with academic stress (*β* = 0.252, *p* < 0.001). For students with average levels of individual academic involution (M), the positive association remained significant but was weaker (*β* = 0.172, *p* < 0.01). For students with high levels of individual academic involution (M + 1SD), the association between academic involution atmosphere and academic stress was no longer significant (*β* = 0.092, *p* > 0.05). These results indicate that individual academic involution weakens the association between academic involution atmosphere and academic stress.

**Table 5:** Simple slope analysis of the moderating effect of individual academic involution.

Individual involution	Path	SD	*t* value	2.5%CI	97.5%CI
High (M + 1SD)	0.092	0.095	0.969	−0.088	0.289
Moderate (M)	0.172	0.066	2.594	0.050	0.312
Low (M-1SD)	0.252	0.050	4.963	0.160	0.358

**Figure 2:**
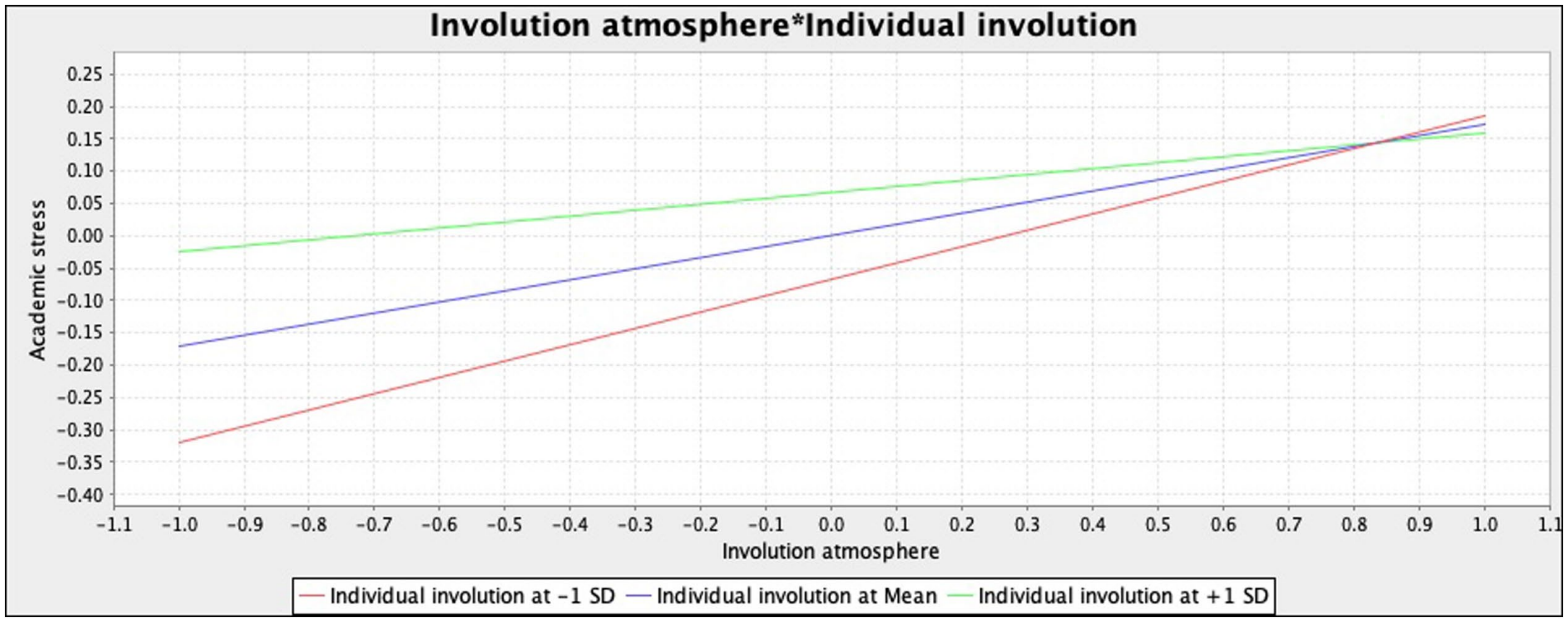
Simple slope analysis of the moderating effect of individual academic involution.

In other words, the higher a student’s individual involution level, the weaker the association between perceived involution atmosphere and academic stress, as illustrated in Figure 2.

## 5 Conclusion and discussion

### 5.1 Conclusion

Guided by concepts from the I-PACE framework and integrating the stress–coping theory, this study proposed a moderated mediation model to examine the association between academic involution atmosphere and AI dependence, with academic stress as a mediator and individual academic involution as a moderator. The results indicate that higher levels of academic involution atmosphere were associated with higher levels of academic stress among students. In addition, academic stress was positively associated with AI dependence. The findings further suggest that the association between academic involution atmosphere and AI dependence was statistically linked to academic stress.

The moderating effect can be primarily understood from a resource-based perspective. Students with higher levels of individual academic involution tend to exhibit stronger self-regulated learning and sustained academic engagement, which may function as important personal resources in managing academic demands. These characteristics may be related to better task organization and sustained academic engagement, which could partly explain the weaker association between external competitive pressure and perceived stress. At the same time, alternative interpretations cannot be fully ruled out. For example, students with high levels of individual academic involution may already experience relatively elevated stress due to strong internal standards and self-imposed competition. In such cases, the weaker association may partly reflect reduced sensitivity to environmental variation rather than purely a weaker association pattern.

### 5.2 Theoretical implications

The findings have several theoretical implications. First, the study enriches the research scope of problematic technology use in the AI era. By focusing on AI dependence as an emerging form of PTU, distinct from traditional internet gaming or social media addiction, the study provides insights into its psychological characteristics and boundary conditions in educational contexts. Unlike traditional forms of problematic technology use that are often associated with entertainment, impulsivity, or social gratification, AI dependence in this study reflects a more academically instrumental and coping-oriented reliance on AI tools.

Second, this research highlights the importance of contextual factors in understanding PTU. While prior studies have primarily focused on individual psychological and personality factors, this study incorporates the social environmental variable of academic involution atmosphere, showing that external competitive contexts are associated with students’ reliance on AI tools. Drawing on social comparison theory, the findings suggest a possible pathway through which competitive environments are linked to perceived pressure, with AI use being associated with coping-oriented responses.

Third, the study highlights the interaction between individual and environmental factors in relation to academic stress and AI dependence. The moderating role of individual academic involution suggests that the association between the academic environment and students’ stress and technology use may vary across individuals with different characteristics. Consistent with the stress–coping perspective, the same external context may be appraised differently, and stressors may be associated with greater psychological burden through cognitive appraisal. These findings contribute to a more nuanced understanding of how personal and contextual factors jointly relate to patterns of AI dependence.

### 5.3 Practical implications

Given the relatively modest effect sizes observed in this study, the following implications should be interpreted as preliminary and exploratory rather than prescriptive recommendations. First, from an educational management perspective, the findings suggest that universities may pay greater attention to students’ perceptions of excessive academic competition. Although the observed associations were modest in magnitude, institutions may consider promoting more balanced learning environments by incorporating diversified assessment approaches (e.g., participation, projects, and collaboration) and encouraging collaborative learning opportunities such as peer learning communities or group-based projects.

Second, the findings suggest that students may respond differently to competitive academic environments. In light of the association between academic stress and AI dependence, universities may consider offering resources related to stress management, self-regulated learning, and adaptive study strategies through workshops, orientation programs, or student support services. The moderating findings also suggest that students with different levels of individual academic involution may perceive competitive environments differently. Students with lower levels of individual academic involution may report relatively higher stress under similar academic conditions. Therefore, universities may consider providing flexible academic support and mentoring resources tailored to diverse student needs.

Third, from an AI literacy perspective, universities may encourage reflective and responsible AI use in academic contexts. Universities may consider providing guidance on appropriate and inappropriate uses of AI tools in learning activities. In addition, institutions can offer workshops or training programs on AI literacy and academic ethics to encourage students to critically evaluate AI-generated content and use AI tools thoughtfully in academic work.

### 5.4 Limitations and prospects

This study has several limitations. First, the cross-sectional survey design captures associations among the variables but does not allow for causal inference. Accordingly, the observed relationships should be interpreted as associative rather than causal. Future research could employ experimental or longitudinal designs to further examine the causal relationships among academic involution atmosphere, academic stress, and AI dependence. Second, the sample consisted entirely of Chinese university students, where academic involution is a relatively salient phenomenon. Future studies could examine whether the proposed relationships generalize to other cultural and educational contexts. Third, the data were collected during the early stage of the academic semester. Because academic stress may fluctuate across different periods of the academic calendar, the findings may partly reflect stress levels specific to this time period. Future research could collect data across multiple academic stages, such as examination or assignment-intensive periods, to further examine the robustness of the proposed relationships under varying levels of academic pressure.

## Data Availability

The datasets presented in this study can be found in online repositories. The names of the repository/repositories and accession number(s) can be found in the article/supplementary material.
